# Changes of cerebral functional connectivity induced by foot reflexology in a RCT

**DOI:** 10.1038/s41598-023-44325-x

**Published:** 2023-10-10

**Authors:** Emeline Descamps, Mathilde Boussac, Karel Joineau, Pierre Payoux

**Affiliations:** 1 Inserm Unité ToNIC, UMR 1214, CHU PURPAN – Pavillon BAUDOT, Place du Dr Joseph Baylac, 31024 Toulouse CEDEX 3, France; 2 CNRS, Toulouse, France

**Keywords:** Neuroscience, Biomarkers

## Abstract

Non-Pharmacological Interventions (NPIs) are increasingly being introduced into healthcare, but their mechanisms are unclear. In this study, 30 healthy participants received foot reflexology (FR) and sham massage, and went through a resting-state functional magnetic resonance imaging (rs-fMRI) to evaluate NPIs effect on brain. Rs-fMRI revealed an effect of both NPIs on functional connectivity with changes occurring in the default-mode network, the sensorimotor network and a Neural Network Correlates of Pain (NNCP—a newly discovered network showing great robustness). Even if no differences were found between FR and SM, this study allowed to report brain biomarkers of well-being as well as the safety of NPIs. In further research, it could be relevant to study it in patients to look for a true reflexology induced-effect dependent of patient reported outcomes. Overall, these findings enrich the understanding of the neural correlates of well-being experienced with NPIs and provided insight into the basis of the mechanisms of NPIs.

## Introduction

Non-Pharmacological Interventions (NPIs) are non-invasive, targeted and evidence-based interventions that aim to prevent, care for, or cure individual’s health problems, as defined by the Non-Pharmacological Interventions Society (NPIS)^1^. Among NPIs, Foot Reflexology (FR) consists of physical stimulation of the epidermis of the feet through the application of controlled pressure movement to specific areas, called reflex zones. This concept was used to activate homeostasis^2,3^ and currently, FR is increasingly being introduced into healthcare to improve the physical and emotional well-being of individuals. Some studies have already reported significant positive outcomes including pain management, regardless of etiology^4–11^, stress or anxiety^12–14^ and improvement in general well-being or quality of life^15–17^. Despite these benefits, research based on robust methodology was sparse and the mechanisms underlying the therapeutic effects of FR also remained undetermined^18^. Hence, clarifying the indications, verifying the safety and identifying the mechanisms of FR is currently a key priority.

For this purpose, functional magnetic resonance imaging (fMRI) is a powerful tool to observe experience-related cerebral changes as brain’s response to a stimulus. Indeed, resting state fMRI (rs-fMRI) on healthy adult volunteers allows researchers to test hypotheses about particular functional networks and the impact of specific activities (for example mindfulness meditation) on intrinsic brain connectivity^19,20^. A few previous studies reported the use of fMRI to detect brain activity in FR and have suggested a correlation between somatosensory cortex activity and the stimulation of specific reflex areas in the feet^21,22^. Nevertheless, to our knowledge, the examination of resting state networks associated with FR has never been done. Such research may provide information about the integrity, organization and changes of major functional systems of the brain^23^. Several networks of interest could be investigated in FR such as the Default Mode Network (DMN)^24^ associated with episodic memory and self-referential processing; the Executive Control Network (ECN); the Salience Network (SN)^25^; and the Sensorimotor Network (SMN) related to the sense of touch^26,27^. Findings from functional neuroimaging studies on these specific networks have great potential to contribute to the understanding of the mechanisms underlying the therapeutic effect of FR.

We hypothesized that networks associated with attention and sensory processing would show specific changes related to FR and tactile stimulation. The aim of the present study was so to determine if two different forms of short tactile stimulation (FR and foot massage as a sham massage of FR) could change the functional connectivity of intrinsic connectivity networks, physiological parameters and well-being in healthy participants.

## Results

### Demographic data, electrophysiological measures and well-being assessment

Fifteen females and fifteen males were included. The mean age was of 30.3 ± 5.7 years old. Table 1 presents all the demographic characteristics at baseline (t0). Every participant (n = 30) from both groups was included in all the analyses.

**Table 1:** Demographic data (t0) and evolution of electrophysiological measures and well-being assessment between t0 and t1.

	Population	Group A (SM)		Group B (FR)		p.value^t^		
		Pre	Post	Pre	Post	Group	Time	Interaction
n *(F/M)*	30 *(15/15)*	15 *(5/10)*	/	15 *(10/5)*	/	/	/	/
Age *(years)*	30.3 ± 5.7	29.3 ± 5.4	/	31.3 ± 6.1	/	/	/	/
Heart rate *(beats pm)*	65.5 ± 10.7	63.8 ± 10.8	56.9 ± 7.5	67.3 ± 10.6	56.0 ± 11.2	0.6	0.0009*	0.4
Respiratory rate *(breaths pm)*	14.1 ± 3.9	15.0 ± 4.2	16.0 ± 4.2	13.1 ± 3.4	14.4 ± 3.3	0.07	0.01*	0.2
Oxygen saturation *(SpO*_*2*_*)*	97.2 ± 1.9	96.9 ± 1.9	96.9 ± 1.6	97.5 ± 1.9	96.9 ± 2.1	0.5	0.06	0.7
Subjective well-being	25.6 ± 2.7	25.7 ± 2.3	26.9 ± 2.1	25.5 ± 3.2	26.7 ± 2.2	0.7	0.007*	0.1

Significant values are in bold.

Results are given in mean ± standard deviation; ^t^statistical tests of differences between groups, time and group × time interaction according to the normality of data; *p < 0.05; *pm* per minute, *SpO2* peripheral oxygen saturation, pre = t0, post = t1.

Concerning electrophysiological measures and well-being assessment, there were no significant differences between groups at t0 for sex ratio, age, heart rate, respiratory rate, oxygen saturation nor subjective well-being (Table 1).

Table 1 presents the evolution of heart rate, respiratory rate and oxygen saturation between t0 and t1 for both groups. There was a significant effect of time (intervention and control) for heart and respiratory rates and subjective well-being (p = 0.0009, p = 0.01 and p = 0.007, respectively) with no effect of groups (interaction: p > 0.05): heart rates decreased in both groups, while respiratory rates and subjective well-being increased in both groups.

### Imaging results

Before conducting our analyses on the whole cross-sectional study, we wanted to make sure that the “washout time” (10 min) between t1 and t2 (after the first intervention and before the second one) was enough for connectivity measures to return to «baseline functional connectivity». Therefore, we looked for differences between t1 and t2 in ROI-to-ROI analyses with the CONN toolbox in each network. No significant changes were observed between t1 and t2 in all networks (DMN, SMN, SN, ECN) or NNCP, while significant connectivity changes were observed between t0 and t2 in DMN, ECN and NNCP. Altogether, these results mean that the potential connectivity change after the first intervention (FR or SM) was probably maintained at t2 and that the short “washout period” (imposed by the constant experimental run in the MRI scanner) was not sufficient to see the effect of the second intervention (either FR or SM) on brain connectivity changes. Hence, regrouping every subject from both groups according to the intervention to look for connectivity changes will not be possible since connectivity measures from the second resting state fMRI acquisition (t2) would not be neutral (not corresponding to the baseline connectivity). Subsequently, only analyses concerning the first block (t0 and t1) of the cross-sectional study were done to avoid evaluating a connectivity “contaminated” (t2–t3) by the preceding intervention (1st intervention, t1).

Hence, the following paragraphs present the global overview of the results for the different networks: the changes of connectivity between t0 and t1 and between groups. Descriptions and statistics of the significant clusters are presented in Table 2.

**Table 2:** Descriptions and statistics of the clusters presenting a significant change of connectivity from t0 to t1 in all participants and in the different studied networks.

Networks	Clusters *(with their composing ROIs)*	Statistic	p-unc	p-FDR
DMN	**Cluster 1** *Posterior Cingulum lr—PreCuneus lr_2* *PreCuneus lr—Posterior Cingulum lr* *PreCuneus lr—PreCuneus lr_2*	F(29) = 14.96	0.000573	0.012023
SMN	**Cluster 2** *Pre- and post-central gyri r—Supplementary motor area r* *Pre- and post-central gyri r—Thalamus l*	F(29) = 7.97	0.008517	0.025550
Neural Network Correlates of Pain (NNCP)	**Cluster 3** *Precentral Gyrus l—Postcentral Gyrus l* *Precentral Gyrus l—Postcentral Gyrus r* *Precentral Gyrus r—Postcentral Gyrus r* *Precentral Gyrus r—Postcentral Gyrus l* *Postcentral Gyrus r—Postcentral Gyrus l* *Precentral Gyrus l—Precentral Gyrus r*	F(29) = 18.50	0.000175	0.004913
	**Cluster 4** *Putamen—Amygdala l* *Anterior Insula l—Hippocampus l* *Anterior Insula r—Hippocampus l* *Anterior Insula r—Amygdala r* *Anterior Insula r—Amygdala l* *Putamen—Amygdala r* *Putamen—Hippocampus l* *Anterior Insula r—Hippocampus r* *Cingulate Gyrus, anterior division—Hippocampus r* *Anterior Insula l—Amygdala r*	F(28) = 8.98	0.000972	0.013612
	**Cluster 5** *Postcentral Gyrus l—Thalamus l* *Precentral Gyrus l—Thalamus l* *Postcentral Gyrus r—Thalamus l* *Postcentral Gyrus l—Thalamus r* *Precentral Gyrus l—Thalamus r* *Precentral Gyrus r—Thalamus l* *Postcentral Gyrus l—VTA* *-Postcentral Gyrus r—Thalamus r* *Precentral Gyrus r—Accumbens r* *Precentral Gyrus r—Thalamus r* *Precentral Gyrus r—Accumbens l* *Postcentral Gyrus l—Accumbens l*	F(28) = 7.47	0.002516	0.023484
	**Cluster 6** *Thalamus r—Thalamus l*	F(28) = 6.01	0.006757	0.047297

*p-unc* p.value uncorrected, *p-FDR* p.value corrected with the False Discovery Rate correction, *l* left, *r* right, *lr* left and right.

### DMN (default mode network)

**Figure 1:**
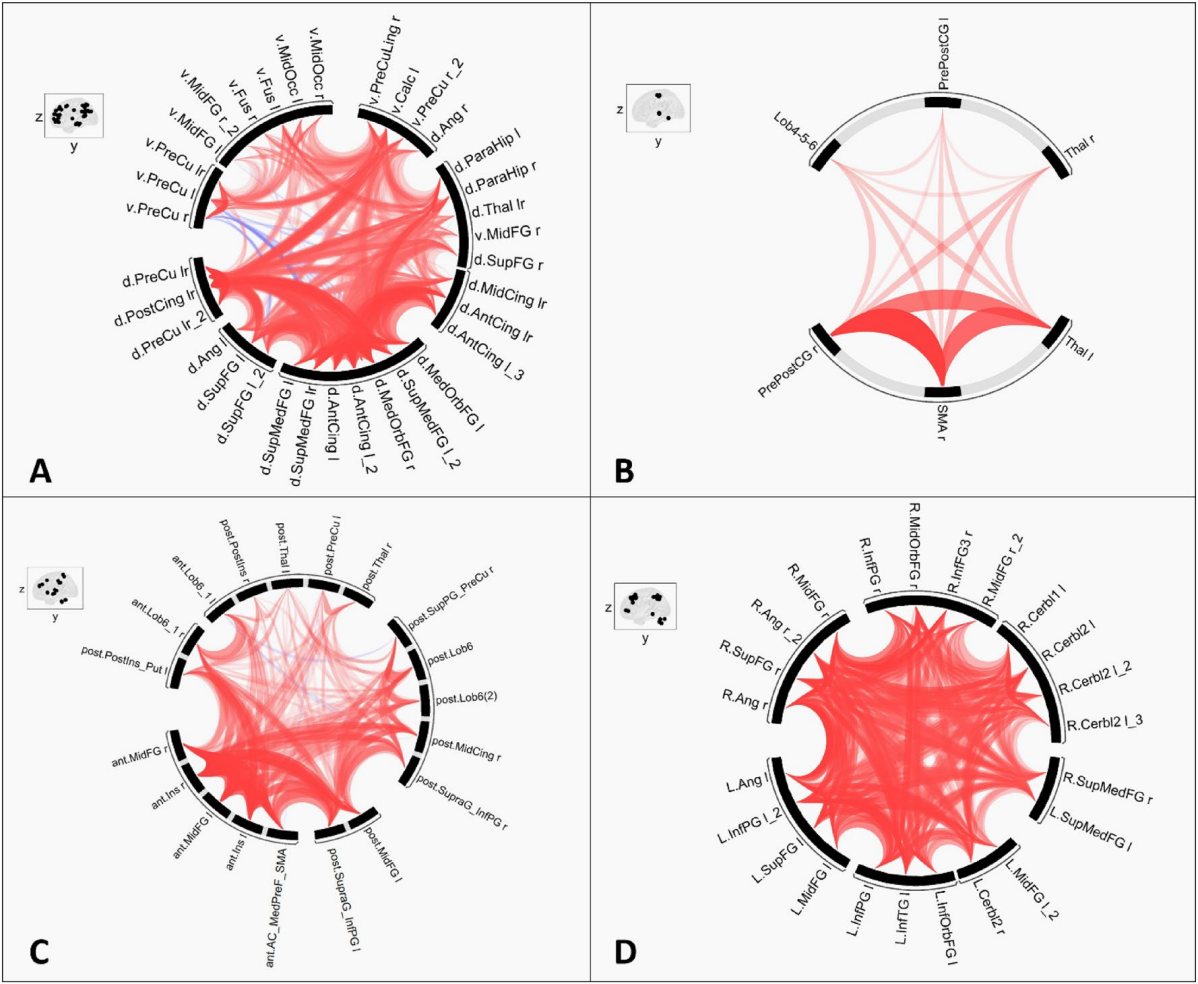
Representations of the connectivity at t0 within the four following networks: Positive correlations in red; negative correlations (anti-correlations) in blue; for a better understanding, thickness and/or opacity of lines are proportional to the statistics of correlations; *l* left, *r* right, *lr* left and right. (**A**) Default Mode Network (DMN)—*d.* dorsal DMN, *v.* ventral DMN, *SupFG* superior frontal gyrus, *SupMedFG* superior medial frontal gyrus, *AntCing* anterior cingulum, *MedOrbFG* medial orbital frontal gyrus, *Ang* angular gyrus, *PreCu* precuneus, *PostCing* posterior cingulum, *MidCing* middle cingulum, *Thal* thalamus, *ParaHip* para-hippocampus, *Calc* calcarine, *MidFG* middle frontal gyrus, *Fus* fusiforme, *MidOcc* middle occipital, *PreCuLing* precuneus/lingual gyrus. (**B**) Sensorimotor Network (SMN)—*PrePostCG* pre- and post-central gyri, *SMA* supplementary motor area, *Thal* thalamus, *Lob4-5–6* bilateral lobules IV, V and VI. (**C**) Salience Network (SN)—*ant.* anterior salience network, *post.* posterior salience network, *MidFG* middle frontal gyrus, *Ins* insula, *AC_MedPreF_SMA* anterior cingulate cortex, medial prefrontal cortex and supplementary motor area, *Lob6_1* lobule VI, crus I, *SupraG_InfPG* supramarginal gyrus and inferior parietal gyrus, *PreCu* precuneus, *MidCing* midcingulate cortex, *SupPG_PreCu* superior parietal gyrus and precuneus, *Thal* thalamus, *Lob6* lobule VI, *PostIns_Put* posterior insula and putamen, *PostIns* posterior insula. (**D**) Executive Control Network (ECN)—*R.* right ECN, *L.* left ECN, *SupFG* superior frontal gyrus, *SupMedFG* superior medial frontal gyrus, *InfOrbFG* inferior orbital frontal gyrus, *MidOrbFG* middle orbital frontal gyrus, *InfFG3* inferior frontal gyrus F3, *MidFG* middle frontal gyrus, *Ang* angular gyrus, *InfPG* inferior parietal gyrus, *InfTG* inferior temporal gyrus, *Cerbl1* crus 1 of the cerebellum, *Cerbl2* crus 2 of the cerebellum.

At t0, the 34 ROIs forming the DMN from the Willard atlas were well connected between them with a ratio of connectivity of 84.8%. There were no differences between groups in the DMN at t0 (Fig. 1A).

**Figure 2:**
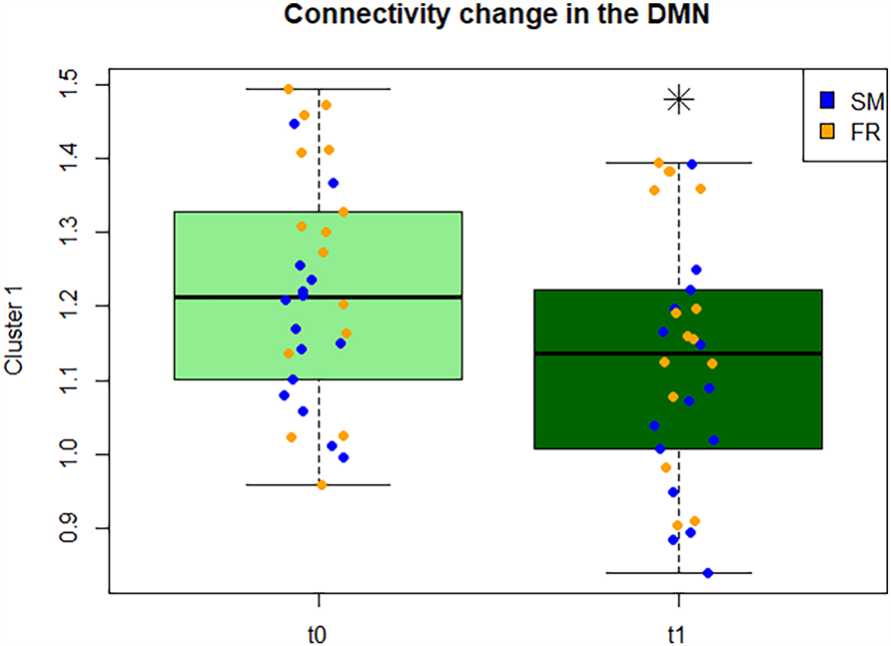
Boxplot of the change of connectivity between t0 and t1 in the DMN in all participants. Dots represent each participant: group A receiving the Sham Massage (SM) in blue and group B receiving the Foot Reflexology (FR) in orange; *p-FDR corrected < 0.05.

Between t0 and t1, a significant change of connectivity was observed in a cluster (cluster 1—Table 2) formed by the posterior cingulate (left and right) and the precuneus (left and right) (p-FDR = 0.01): there was a diminution of connectivity in this cluster 1 after both interventions (FR and SM) (Fig. 2).

There were no differences of change according to groups.

### SMN (sensorimotor network)

At t0, the 6 ROIs forming the SMN from the Willard atlas were well connected between them with a ratio of connectivity of 100%. There were no differences between groups in the SMN at t0 (Fig. 1B).

Between t0 and t1, a significant change of connectivity was observed in a cluster (cluster 2—Table 2) formed by the right pre- and post-central gyri, the right supplementary motor area and the left thalamus (p-FDR = 0.02): there was an augmentation of connectivity in this cluster 2 after both interventions (FR and SM) (Supplementary Fig. 1).

There were no differences of change according to groups.

### SN (salience network)

At t0, the 19 ROIs forming the SN from the Willard atlas were well connected between them with a ratio of connectivity of 84.2%. There were no differences between groups in the SN at t0 (Fig. 1C).

Between t0 and t1, there was no significant change of connectivity in this network, even when considering the groups effect.

### ECN (executive network)

At t0, the 23 ROIs forming the ECN from the Willard atlas were well connected between them with a ratio of connectivity of 97.6%. There were no differences between groups in the ECN at t0 (Fig. 1D).

Between t0 and t1, there was no significant change of connectivity in the ECN, even when considering the groups effect.

### Neural Network Correlates of Pain (NNCP): 23 ROIs associated with pain

**Figure 3:**
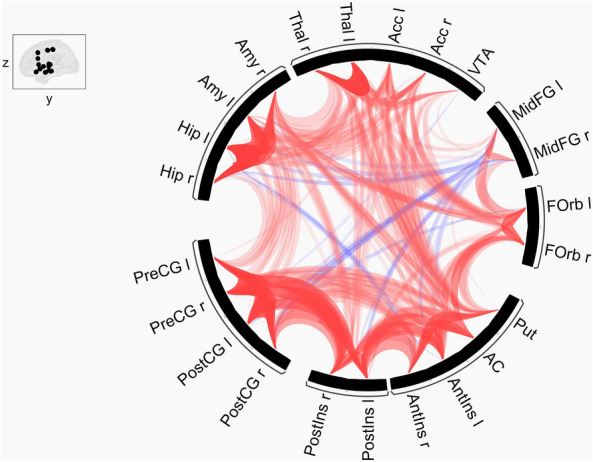
Representation of the connectivity within the NNCP at t0. Positive correlations in red; negative correlations (anti-correlations) in blue; for a better understanding, thickness and/or opacity of lines are proportional to the statistics of correlations; *l* left, *r* right, *PreCG* precentral gyrus, *PostCG* postcentral gyrus, *PostIns* posterior insula, *AntIns* anterior insula, *AC* cingulate gyrus, anterior division, *Put* Putamen, *Forb* frontal orbital cortex, *MidFG* middle frontal gyrus, *VTA* ventral tegmental area, *Acc* accumbens, *Thal* thalamus, *Amy* amygdala, *Hip* hippocampus.

At t0, the 23 ROIs selected for their implication in pain were well connected between them with a ratio connectivity of 74.7%. Therefore, it seems reasonable to consider these 23 ROIs as a NNCP. Moreover, there were no differences between groups in this NNCP at t0 (Fig. 3).

Between t0 and t1, significant changes of connectivity were observed in four clusters (Table 2): (1) cluster 3 was formed of pre- and post-central gyri (left and right); (2) cluster 4 consisted of the putamen, the anterior cingulate gyrus, the amygdala, the anterior insula and the hippocampus (all in their left and right sides); (3) cluster 5 comprised the pre- and post-central gyri, the thalamus, the accumbens nucleus (all in their left and right sides) and the VTA; and (4) cluster 6 was made of the both thalami (left and right). Hence, after the interventions (FR or SM), there were an augmentation of connectivity in clusters 3, 4 and 6 (p-FDR = 0.0003, p-FDR = 0.0001 and p-FDR = 0.0009, respectively), and a diminution of connectivity in cluster 5 (p-FDR = 0.0003) after both interventions (FR and MS) (Supplementary Fig. 2).

There were no differences of change according to groups.

### Correlations between changes in connectivity values and in electrophysiological measures or well-being assessment after the intervention

As regard to the previous significant results, correlations between percentages of change of connectivity—from the DMN cluster, the SMN cluster and the four clusters of the NNCP—and the percentages of change of heart and respiratory rates and subjective well-being were evaluated. Only one correlation was significant, before adjustment for multiple analysis: there was a negative correlation between change of connectivity in the cluster 5 of the NNCP and change of the respiratory rate (Spearman correlation: p = 0.03 and rho = − 0.40). More the connectivity of the cluster 5 diminished, higher was the respiratory rate of the participants.

### Safety of non-pharmacological Interventions

No adverse events and no discomfort were reported by participants during the research protocol.

## Discussion

This study first assessed the impact of Non-Pharmacological Interventions (NPI) on brain connectivity in well-known cerebral networks (the DMN, the SMN, the SN and the ECN) and in a newly investigated Neural Network Correlates of Pain (NNCP) of healthy volunteers. We showed that NPIs (FR and SM) led to changes of connectivity in the DMN, SMN and NNCP. Nonetheless, no specific effect of FR was found in this study compared to the SM, for one unique 10-min session. Furthermore, well-being was improved in the participants after both NPIs (FR and SM), as seen through the subjective assessment (questionnaire) and objective electrophysiological measurements (heart and respiratory rates). This increase in well-being could be related to the connectivity changes. Moreover, no adverse events and no discomfort were reported by participants during the research protocol. Thus, it seems reasonable to consider FR and SM safe in this context.

Firstly, non-Pharmacological Interventions influence some cerebral networks. Indeed, the first result of our study is that tactile foot touch (FR and SM) has an effect on functional connectivity, specifically in some well-known networks, the DMN and the SMN, and in our newly investigated NNCP. Primarily, within the DMN, there was a diminution of connectivity between the posterior cingulate and the precuneus (cluster 1). The posterior cingulate cortex is known as the central node of the DMN, which deactivates during externally attentional tasks or meditation^28,29^. Hence, this decreased connectivity may indicate that tactile foot touch (FR or SM) influence the state of consciousness of the participants and lead them to a state of more focused attention. Indeed, Vogt and collaborators (2005) stipulated that the posterior cingulate and precuneus cortices are necessary to conscious information processing, through a decrease of activity, and are part of a so-called “neural correlates of consciousness”^30^. Secondary, within the SMN, there was an augmentation of connectivity between the right pre- and post-central gyri, the right supplementary motor area and the left thalamus (cluster 2). The pre- and post-central gyri are composed of the primary motor cortex and the primary somatosensory cortex respectively. The primary somatosensory cortex being the main sensory receptive area for the sense of touch^31^, the increased connectivity between the pre- and post-central gyri is coherent with the sensory features of the NPIs sensed by the participants. Indeed, only the right parts of these pre- and post-central gyri have shown a change of connectivity while tactile foot touch (FR or SM) were on the left foot (brain contralateral activation). Similarly, only the contralateral part of the supplementary motor area (SMA) was affected—the SMA responding both to motor and sensory tasks^32^. Concerning the connectivity with the left thalamus, which is implicated in motor and sensory features, a study have demonstrated that stimuli applied to the left side of the body led to responses in some left and right nuclei of the thalamus^33^.

The change of connectivity in the NNCP is our second main new result. Interestingly, we found that functional connectivity from the NNCP increased in the anterior cingulate gyrus, the anterior insula, the thalami and the pre- and post-central gyri (clusters 3, 4 and 6) after tactile foot touch, while it became anti-correlated in a cluster formed by the thalami, pre- and post-central gyri and the VTA (cluster 5). These results seem coherent with the literature on other NPIs. Indeed, a previous study has shown changes of functional connectivity in the midcingulate cortex, the insula, the pregenual, frontal regions and the pre-SMA (Supplementary Motor Area) after hypnosis, a different NPI^34,35^. In the cited study, the increase of connectivity was also correlated with the reduction of pain^34^. In the present study, we also observed an augmentation of connectivity between the pre- and post-central gyri (cluster 4), as seen in the SMN, but, this change may be unspecific to pain and rather related to sensory features, as explained before. Similarly, the insula has been shown to be related to pain consciousness but not in a specific matter^36^. Indeed, it is thought that the insula participates in the perception of magnitude of different sensorial stimuli, such as the touch. Hence, the change of connectivity in cluster 3 involving the insula could also be due to the tactile stimulation during the two NPIs (FR and SM). The thalamus is implicated in many systems since it appears to be a relay center of lateral and median pathways transmitting neural signals to the cerebral cortex from a number of brain areas^37^. Indeed, the thalamus is implicated in the ganglia-thalamo-cortical circuit involved in movement^38^, as well as in chronic pain^39^ etc. For sake of clarity, our study is not sufficient to stipulate the role of NPIs such as FR in NNCP; however, this was not the aim of the study. We investigated healthy volunteers without any pain symptoms to show that tactile foot touch had an effect on cerebral connectivity, which we managed successfully, and to propose the existence of a NNCP since relevant functional connectivity was found at baseline in our selected brain regions. In our study, we report an amelioration of participants’ well-being. As they were healthy volunteers, we cannot conclude about a modification of pain perception in our participants after tactile foot touch. More studies on this NNCP are needed to better assess its involvement in pain and pain management in patients with chronic pain conditions, such as Parkinson’s disease, to cite an example.

**Figure 4:**
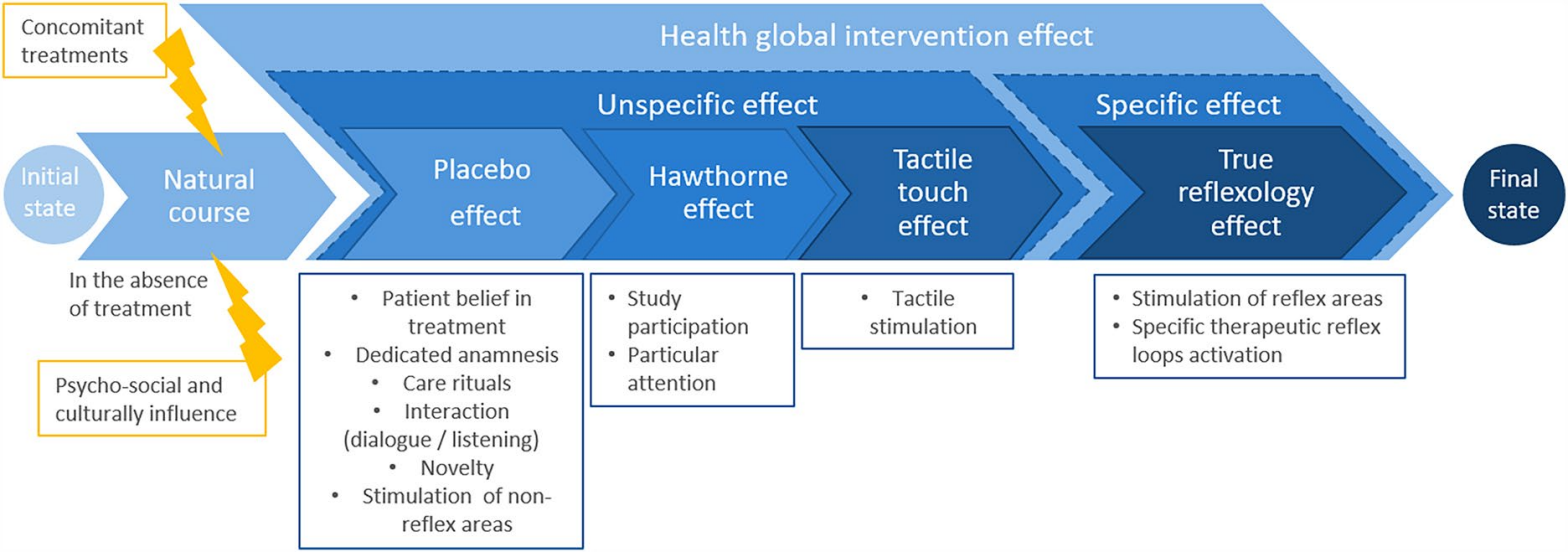
Summary chart of factors influencing reflexology intervention outcomes.

Secondly, both foot reflexology and sham massage contribute to participants’ well-being. In our study, we did not find any difference between FR and SM. The absence of specificity of FR could be explained by the characteristics of our participants: healthy volunteers in no need of cure. In this paradigm, the well-being was a health component (Fig. 4). Therefore, we hypothesize that FR improves well-being in healthy participants as well as SM, and that FR specificity might only be apparent in cure management of patients. This hypothesis was suggested by Boitout & Vadala, who argued that specific therapeutic reflex loops would only exist in the presence of pathological reflex loops associated with dysfunctions and podal reflex zones^40^. Indeed, in our study, electrophysiological measures and subjective evaluations have shown that participants’ well-being increased similarly after both tactile foot touches. In this notion of well-being, it has been also shown that reflexology induces relaxation and a cerebral activity similar to the one seen in a sleep state^41^, as well as to foot massage, which induces relaxation and increases resting-state alpha activity^42^. Along similar lines, a study has shown that true acupuncture (another NPI) induced the same fMRI changes (hemodynamic response) than sham acupuncture in healthy adults^43^. Therefore, in healthy subjects, both sham interventions (SM as well as sham acupuncture) seem as much efficient as the true interventions (FR or acupuncture). In another study comparing stroke patients to healthy subjects, true acupuncture also led to changes of effective connectivity in both groups, but some of these changes were different between groups^44^. Hence, our results observed here on healthy volunteers may be different from a true reflexology induced-effect which could be seen in patients reported outcomes.

Moreover, in NPIs, it is difficult to separate the specific effect of the carried intervention from all the unspecific effects (Fig. 4). Indeed, from the initial to the final state, several factors influence the measured outcome. First, there is the well-known placebo effect, which depends on the belief, expectations and needs of the patient/participant in the treatment/intervention. Second, there are the relationship with the practitioner of the NPI, the patient’s personality^45^, and the ritual, in the case of FR or SM, on the stimulation of non-reflex areas^45,46^. Additionally, there is the Hawthorne effect^47^, which depends on the volunteer motivation to participate to the study and its will to please the practitioner. The specific effect of the NPI (FR here) appears only then, due to the stimulation of the reflex areas^18^. Hence, in our study on healthy volunteers, all the unspecific effects present in the SM may have been sufficient to induce a state of well-being, especially since human touch has been proved “to promote healing and bring comfort”^18^, and therefore sufficient to lead to brain connectivity changes. Furthermore, in this study, FR was performed on the reflex area associated with the diaphragm, which regulates heart and respiratory rates and emotional stress related to pain. The diaphragm reflex area has a role in the well-being such as the SM. That could explain why similar results were found with both NPIs in terms of connectivity and electrophysiological changes^48–50^.

Thirdly, our aim—through the study of a pain-related network (the Neural Network Correlates of Pain (NNCP))—was to investigate the existence and the robustness of this network in healthy volunteers. Moreover, it is known that pain perception arises through complex interconnection inside the brain and that some cortical regions related to pain become active simultaneously during normal pain perception in healthy individuals^39^. This normal activation could therefore be associated with some functional connectivity network. Hence, we have shown here, even in the absence of painful stimuli, that this NNCP seems robust to functional analyses. Indeed, even before the NPIs, our study has revealed the robustness of a NNCP formed of 23 ROIs (with left and right hemisphere division): its density of connectivity was of 74.7%, which seems to legitimise its qualification as a NNCP. Thus, we were able to spot this NNCP in healthy volunteers which is very interesting since, according to the literature, even in the absence of pain, some brain connections related to pain may still be active^51^. These results provide so benchmarks in healthy volunteers, which could serve as a foundation for future studies in patients. Therefore, our study paves the way to future researches in the field of pain management to better understand the functioning of this new NNCP and verify its correlations with pain (pain intensity, acceptance of pain, sensory-discriminative and affective components of pain etc.). To this end, it appears crucial to test the specific effect of FR on chronic pain, for example in Parkinson’s disease patients through this new paradigm.

The main limit of this work would be the brevity and the single occurrence of the FR and SM interventions, which is not representative of the effects that might be experienced in a reflexology or tactile foot touch setting (typically more than one session). The scanning procedure was limited in order to reduce the discomfort of participants in this restrictive environment. Thus, the interventions were only applied to the left foot in order to obtain reliable neural activity response. As well, another important limitation of this study includes the absence of pain evaluation (since our study was done on healthy volunteers) necessary to confirm the involvement of our newly investigated NNCP in pain sensations.

The main strength of our study is the establishment of a robust protocol of FR which induced cerebral functional connectivity changes at rest and therefore could be used in others studies on clinical care. Our protocol is reproducible which is important to create more studies in personalized medicine approaches^18^. Indeed, we described precisely "what is done" during the tactile foot touch, "what is experienced" and "what does it feel like" to receive these interventions, in order to identify biomarkers for these changes, to use subjective and objective criteria of judgment. Moreover, it seems that using SM as control would be a good approach to separate at best the specific claimed effect of reflexology which is very important for a qualitative analysis of reflexology effects^18^. Finally, both interventions were performed by the same researcher to avoid any bias related to the influence of practitioner interaction or individuality.

## Methods

The challenge of the FOOT study was to measure the immediate impact of tactile stimulations on various subjective and objective parameters. Indeed, there are some probabilities that not only the specific condition, but also a variety of baseline conditions change during the experimental condition^52^. Thus, we have chosen to carry out the entirely study in the MRI scanner, to avoid any unnecessary movement etc.

### Study design

FOOT was a randomized double-blinded (participants and interpretation) prospective monocentric study. It was cross-sectioned so that each volunteer received both the intervention (foot reflexology—FR) versus the control (foot massage as a sham massage of FR—SM).

The study was registered in ClinicalTrials.gov Identifier (NCT04661774—10/12/2020) after the study was approved by the French Ethic Committee (personal protection committee: CPP Ile de France III n°3827-RM) on September 08, 2020, and all methods were carried out in accordance with relevant guidelines and regulations. Then, all volunteers were included in the study between February and June 2021 after giving their written informed consent, in the ToNIC laboratory in Toulouse, France. The 30 volunteers completed the entire study.

Healthy volunteers (n = 30) between 20 and 40 years old were included. All volunteers were right-handed, attested by a score superior or equal to 8/10 at the Edinburgh handedness inventory^53^, and naïve to FR. They had no MRI contra-indications, nor neurodegenerative or cardiac pathologies and no injuries in the left foot, on which the FR or SM were performed.

Randomisation was established with the help of the MeDatAS unity from the Clinical Investigation Center (CIC) of Toulouse. Once included, participants were affected to a number and a group through a secure randomization interface via the CIC website. Volunteers were randomly split into two groups of 15. Group A received first the SM and secondly the FR; while group B received first the FR and secondly the SM, in order to see both the true FR and SM effects despite potential individual differences.

**Figure 5:**
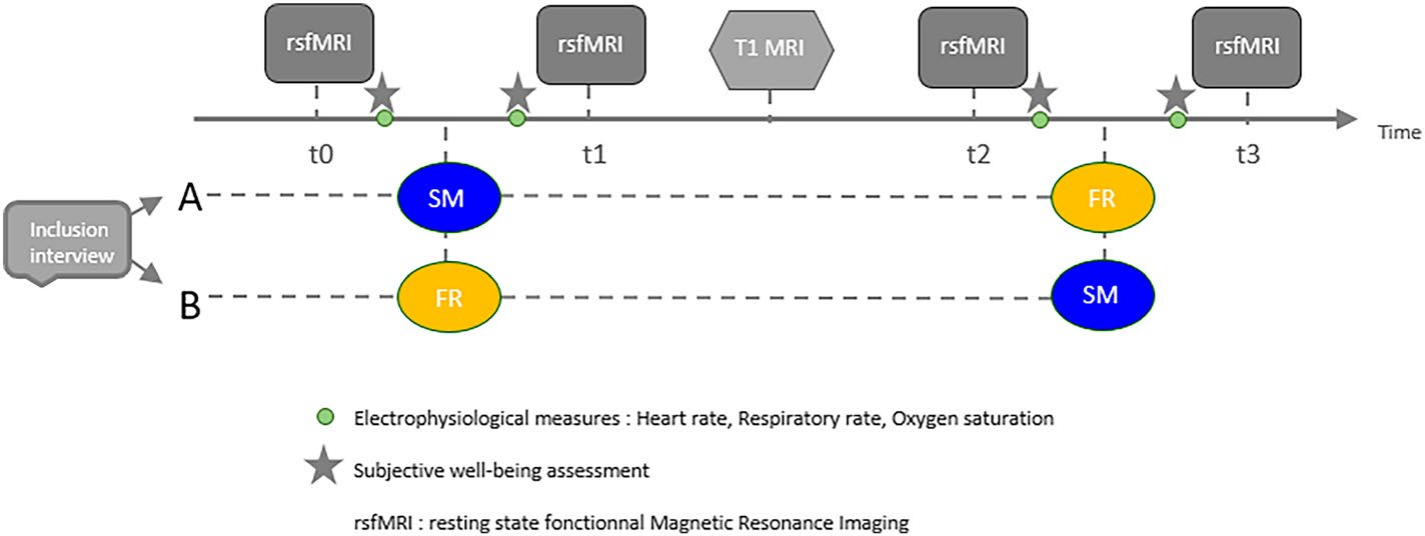
Study design. *SM* Sham Massage, *FR* Foot Reflexology.

After inclusion, resting-state functional MRI (rs-fMRI) acquisitions were done before (t0) and after (t1) the first intervention or control, depending on the group. The anatomical MRI (T1) acquisition was done in the middle of the cross-sectioned design, to be used as a washout time, that is a break time before the second intervention. Then, rs-fMRI acquisitions were again done before (t2) and after (t3) the second intervention or control, as opposed to the first time. Before and after each intervention and control, electrophysiological measures and a short interview about subjective well-being were done. Study design is presented in Fig. 5.

### Interventions

Both interventions were performed only in the left foot by the same researcher (E.D.), certified in reflexology.

#### Foot reflexology (FR) protocol

**Figure 6:**
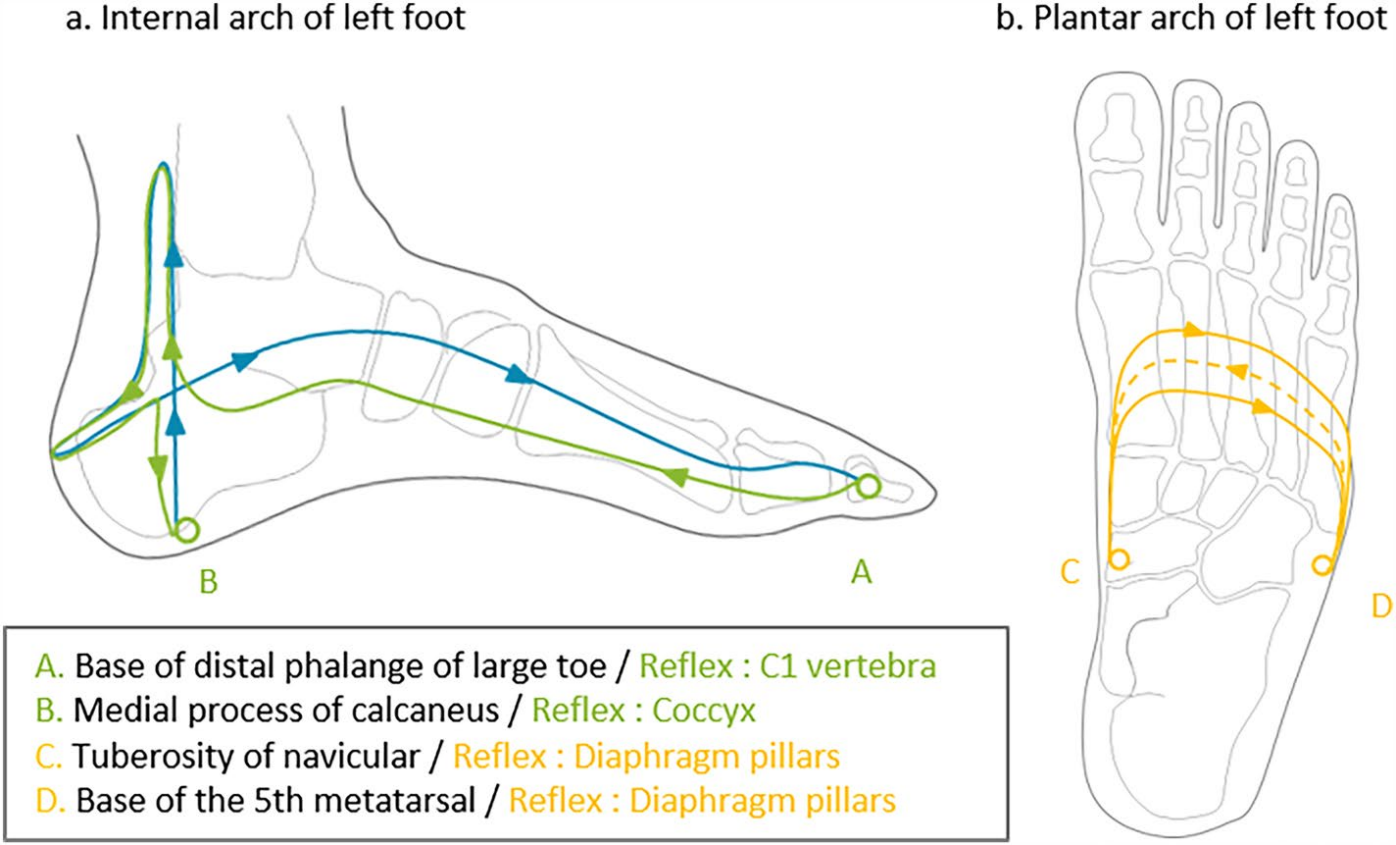
Schema of the reflexology protocol and the stimulated reflex zones. For the spine reflex: The pathway followed is from point A to point B—green, then from point B to point A—blue. For the diaphragm reflex: The pathway followed is from point C to point D—in orange, on the plantar side, and then from point D to point C, on the dorsal side, which is like a spiral around the foot.

The specific method employed here was detailed by Martine Faure Alderson^54^ or Guy Boitout & Jean-Pierre Vadala^40^. It consisted of a specific, non-aggressive and precise solicitation of the cutaneous mechanoreceptors based on body landmarks of the foot map. The technique is exclusively manual, practiced with the pulp of the thumb or index finger. The pressure of the finger is vertical to the skin, in the order of 1 to 3 mm, the depth limit is perceived at the moment when the vertical action of the finger meets a sensation of rebound. A particular movement is used, in which the tip of the working finger travels over the surface of the foot with a movement which can be likened to a "caterpillar crawling" with a "rotation" at the same time, used by most reflexologists^55,56^. This precise and fluid touch allows application of pressure without inducing pain. This approach is based on our experience of working with patients with complex health conditions, pain and altered pain perception. Participants received a standardised 10-min reflexology treatment directly in the MRI scanner. The FR protocol consisted of the application of a special touch to the reflex points of the foot^55^. The zones of stimulation were the reflexes of the spine, which stimulate the nervous system, responsible for maintaining homeostasis and the diaphragm reflex, as shown in Fig. 6, ^6,55–57^.

#### Sham massage (SM) protocol

The sham massage method used here was similar to foot modelling for well-being purposes and aimed to relax the muscles. The participants received a standardised 10-min massage directly in the MRI scanner. The technique is exclusively manual, practiced with the palms of the hands and the fingers. The pressure is vertical to the skin, such as a gentle touch manipulation, the depth limit is before the sensation of rebound. The sham massage protocol consisted of gentle touch of the foot, starting from the toes to the heel, then circling the ankle and moving up to the toes. This sequence was repeated for 10 min. No specific points were pressured, as opposed to FR.

### Electrophysiological measures and well-being assessment

Three electrophysiological measures were recorded before and after each intervention and control, directly inside the MRI scanner, without moving the volunteer: the respiratory rate, the heart rate and the oxygen saturation.

Subjective well-being was assessed using a short-form of four questions rated with a Likert-scale from 1 to 7 (1 being “totally agree” and 7 being “not agree at all”). The questions were: (1) I feel nervous or stressed; (2) I feel relaxed; (3) I have a feeling of pressure in the thoracic cavity; and (4) My breathing is fluid. Total score was calculated by sum of each question score, with an inversion score for questions 2 and 4. Higher is the total score of subjective well-being, better feels the volunteer.

### Imaging acquisition and analyses

MRI data was acquired with an MRI 3T Philips ACHIEVA scanner using a dStream head coil (32 channels). The EPI (Echo Planar Imaging) was used for the fMRI. Structural MRI included an acquisition whole-head in 3 dimensions, in sagittal section and weighted in T1 with the following parameters: MPRAGE, repetition time (RT)/echo time (ET) = 7.4/3.5 ms, flip angle = 8°, field of view (FOV) = 240 × 240 × 180 mm, isotopic voxel size = 1 mm^3^. Functional imaging was done with a pulse sequence weighted T2* echo-gradient (EPI) with the following parameters: RT/ET = 2000/30 ms, flip angle = 90°, FOV = 240 × 240 × 123 mm, isotopic voxel size = 3 mm^3^, 41 jointed axial sections, slice thickness = 3.0 mm, matrix = 80 × 78, 300 volumes, for a whole duration of 10.8 min.

During MRI acquisition, participants were in supine position, the arms to the sides. They were instructed to keep their eyes opened, remain still, relax and not think of anything in particular while staying awake.

Imaging analyses were done with the CONN toolbox from the Massachusetts Institute of Technology (version 21a) functioning under MatLab (version R2017b). Experimental matrix was defined with the four times of evaluation (t0, t1, t2 and t3). Usual pre-treatment was made with the following steps: (1) realignment, (2) slice timing, (3) structural segmentation in grey and white matters and normalization, (3’) functional normalization of the functional images on the MNI (Montreal Neurological Institute) atlas, (4) identification of aberrant activities, and (5) smoothing. Denoising was done through a linear regression of potential confounding effects in the BOLD signal and a default band-pass filter between 0.008 and 0.09 Hz.

An a priori analysis method [Seed Based Analysis (SBA)] was chosen for different ROIs. First, ROIs forming different networks from the Willard atlas, available from the “Functional Imaging in Neuropsychiatric Disorders Lab”, were used. Studied networks were: the Default-Mode Network (DMN), the sensorimotor network (SMN), the Salience Network (SN) and the executive network (ECN). ROI-to-ROI analysis were done to evaluate change inside these networks following interventions. Moreover, 13 ROIs associated with pain were selected from the literature^58–62^: (1) orbitofrontal cortex, (2) dorsolateral prefrontal cortex, (3) anterior cingulate cortex, (4) posterior insula, (5) anterior insula, (6) putamen, (7) nucleus accumbens, (8) hippocampus, (9) amygdala, (10) ventral tegmental area (VTA), (11) thalamus, (12) postcentral gyrus, (13) precentral gyrus. Each ROIs (except the VTA, the putamen and the anterior cingulate cortex) were separated according to their left and right side, leading to a total of 23 ROIs. For these ROIs, we used the coordinates already implanted in the CONN toolbox, which are from the Harvard–Oxford atlas. Only the coordinates from the insula divided into its posterior and anterior regions were taken from the literature (MNI: right posterior insula =  + 39 − 15 + 08; left posterior insula = − 39 − 15 + 01 ; right anterior insula =  + 32 + 16 + 6 ; left anterior insula = − 32 + 16 + 6)^63^, as well as the VTA (MNI: 0 − 15 − 12)^64^. These 23 ROIs were used to test their interactions in ROI-to-ROI analyses, in order to study a potential change of connections in a “new” Neural Network Correlates of Pain (NNCP). This “new” NNCP was created to study effect of NPIs on different networks that our team is interested in, in order to be used afterward in other studies on pain management, if this NNCP proves to be interesting. Indeed, a pilot study on healthy volunteers appeared to be indispensable before proceeding to research in patients’ populations.

### Statistical analyses

R Studio software version 2022.02.3 was used: R Core Team (2022). R: A language and environment for statistical computing. R Foundation for Statistical Computing, Vienna, Austria. https://www.R-project.org/.

The exploratory nature of this study did not allow a sample size calculation, without available data from the literature. A sample size of 30 participants was so defined as the minimum size necessary to observe some significant effect, since it is the commonly used effective in the neuroimaging literature^65,66^.

#### Descriptive statistics and analysis of the electrophysiological measures and well-being assessment

For the demographic data, mean ± standard deviation were calculated for each group, as well as effective and percentage for the qualitative data.

To compare both groups at baseline, the normality of data was first assess using Shapiro tests. If normality was respected, two-sample t-tests of Student were used, otherwise two-sample Mann–Whitney–Wilcoxon tests were done. For qualitative variables, Chi-squared tests were used.

To evaluate evolution of variables after the intervention (FR or SM), ANOVAs were done for the group, time and interaction between group and time, when normality was respected. Otherwise, one-sample Mann–Whitney–Wilcoxon tests for group and for time, and Kruskal–Wallis tests for the interaction between group and time were done.

#### Imaging analyses

Concerning the imaging analyses, only ROI-to-ROI analyses were used in the CONN toolbox, and post-hoc analyses and visualisations were done by extracted the connectivity values into an extern statistical software *(R Studio version 2022.02.3)*.

First, the existence and robustness of each studied network were tested at baseline (t0) in all participants (linear model). A density of connectivity within each network (ratio of connectivity: number of found connections/number of possible connections) superior or equal to 70% was consider as relevant for a functional network, since it is usually the threshold used to indicate a strong relationship in a correlation matrix^67^. Hence, a density of connectivity superior or equal to 70% would represent a dense network with homogeneous connections between each node forming the network.

Secondly, effect of group (A > B) was tested at baseline (t0) to confirm that there was no difference between groups at baseline, one again in each studied network (t-tests).

Then, changes of connectivity after the first intervention (t0 > t1) inside each studied network were evaluated in all participants to look for a modification of network-connectivity caused by the intervention or control (FR or SM) (paired t-tests).

Afterwards, changes of connectivity after the first intervention (t0 > t1) inside each studied network were evaluated between groups (A > B) to look for a group-effect in the change of connectivity of each network (ANOVAs). If significant results were found in the CONN toolbox, connectivity values of the concerned clusters of ROIs were extracted and used for post-hoc analyses.

Finally, percentages of changes of connectivity ((t1 – t0)/t0 × 100) were calculated from the connectivity values of the clusters that changed after the intervention, as well as percentages of evolution of the electrophysiological measures and well-being assessment that significantly change after the intervention or control. Correlations were evaluated between these percentages (percentages of changes of connectivity and percentages of physiological changes) using Pearson or Spearman correlations, according to normality of data.

The same previous analyses were planned for the second intervention (t2 vs t3).

### Supplementary Information

Supplementary Figures.

## Data Availability

The datasets generated and analysed during the current study are not publicly available since data sharing outside the study lab was not included in the informed consent and as such we cannot share the data used for this study, but they will be made available from the corresponding author on reasonable request.
