# Supplementary material for: Changes of cerebral functional connectivity induced by foot reflexology in a RCT
Source: Sci Rep. 2023 Oct 10;13:17139. doi: 10.1038/s41598-023-44325-x (PMC10564852; doi:10.1038/s41598-023-44325-x)
Supplement: Supplementary file 1 — Supplementary Figures. [file 41598_2023_44325_MOESM1_ESM.docx]

*
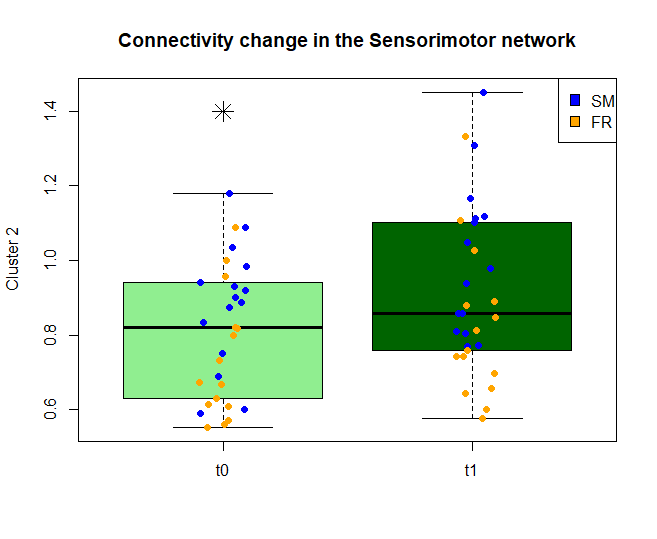
*

*Supplementary Fig. 1 – Boxplot of the change of connectivity between t0 and t1 in the SMN in all participants*

*Dots represent each participant: group A receiving the Sham Massage (SM) in blue and group B receiving the Foot Reflexology (FR) in orange; * p-FDR corrected < 0.05*


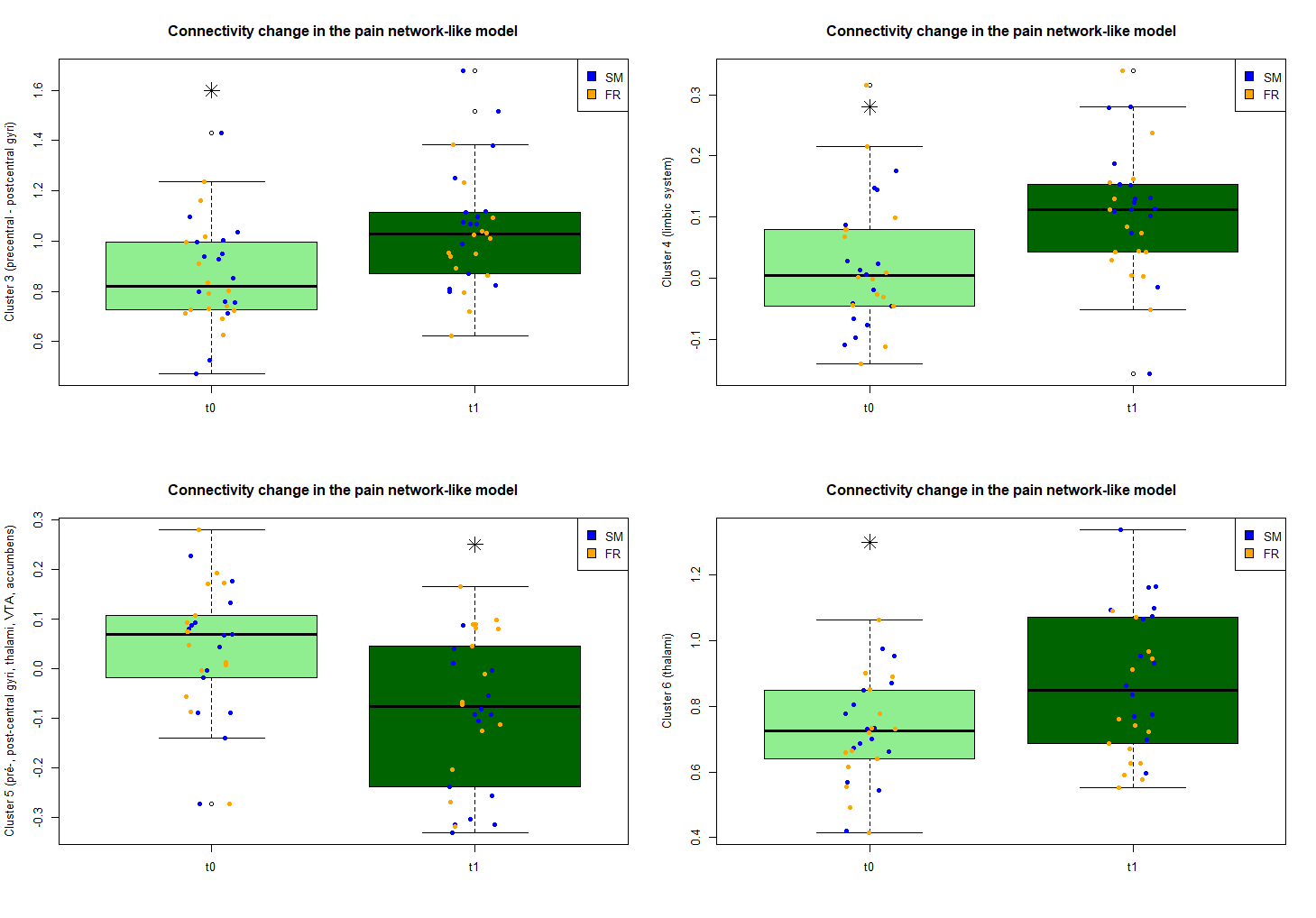


*Supplementary Fig. 2 – Boxplot of the change of connectivity between t0 and t1 for the four clusters in the Neural Network Correlates of Pain (NNCP) in all participants*

*Dots represent each participant: group A receiving the Sham Massage (SM) in blue and group B receiving the Foot Reflexology (FR) in orange; * p-FDR corrected < 0.05*
